# Esophagitis in Children with Celiac Disease

**DOI:** 10.5402/2011/489065

**Published:** 2011-07-13

**Authors:** Wael N. Sayej, Razan AlKhouri, Robert D. Baker, Raza Patel, Susan S. Baker

**Affiliations:** ^1^Digestive Diseases, Hepatology and Nutrition Center, Connecticut Children's Medical Center, School of Medicine University of Connecticut, Framington, CT 06030, USA; ^2^Digestive Diseases and Nutrition Center, Women and Children's Hospital of Buffalo, University at Buffalo, NY 14222, USA

## Abstract

*Objectives.* To our knowledge, the occurrence of esophagitis in children with celiac disease (CD) has never been evaluated. The aim of this study is to determine the prevalence of esophagitis in children with CD. *Patients and Methods.* Between 2003 and 2007, children with biopsy confirmed CD were retrospectively identified. Biopsy reports were reviewed for esophageal inflammation. Biopsy reports of 2218 endoscopies performed during the same period were also evaluated for inflammation. *Results.* Forty-nine children diagnosed with CD (47% boys). Nineteen of 49 (39%) patients with CD had esophagitis (95% CI 0.23–0.5). Thirty percent of boys and 46% of girls with CD had esophagitis (95% CI 0.12–0.40). Overall, 45% of patients who underwent upper endoscopy had esophagitis. The prevalence of esophagitis in CD (39%) compared to the prevalence of esophagitis (45%) in our practice was not significantly different, *P* = 0.2526. *Conclusion.* There was no difference in the prevalence of esophagitis between children diagnosed with CD at the time of their diagnostic EGD and the prevalence of esophagitis in children without CD. A prospective study to determine whether the esophagitis should be treated with acid suppression or whether the esophagitis heals with the gluten-free diet is warranted.

## 1. Introduction

Celiac disease (CD) is an immune-mediated disease found in genetically susceptible people of North American, European, and Middle Eastern backgrounds. Exposure to gluten (wheat, barley, and rye) initiates an inflammatory cascade leading to intestinal injury. CD is histologically characterized by blunting of the villous structures and the presence of intraepithelial lymphocytes in the small intestines. The increase in the estimated prevalence of CD in children from 1 : 1000 to 1 : 3000 to 1 : 100 is likely due to the availability of serologic markers, increased screening, and recognition of vulnerable populations such as Down's Syndrome and type one diabetes [1–3].

The prevalence of esophagitis in children with known gastroesophageal reflux disease (GERD) is estimated at 15–62% [4–6] and there can be an overlap in the symptoms associated with GERD and CD. The coexistence of esophagitis in children with histologically confirmed CD has not been evaluated. The aim of this study is to determine the prevalence of esophagitis in children with CD.

## 2. Materials and Methods

### 2.1. Patients

To determine the prevalence of esophagitis in patients with CD, the records of all patients who underwent esophagogastroduodenoscopies (EGD) with biopsies at the Women and Children's Hospital of Buffalo, University at Buffalo, between January 1, 2003 through December 31, 2007, were searched for the diagnosis code 579.0 (Celiac Disease). To determine the prevalence of esophagitis in children undergoing EGD with biopsies, all records of EGD from January 1, 2003 through December 31, 2007 were reviewed. Those who had mucosal biopsies at the time of endoscopy were included in the analysis. Patient demographics, laboratory data, clinical presentation, histology, and endoscopy results were retrieved from office charts, pathology reports, and electronic records.

### 2.2. Upper Endoscopy with Biopsies

The indications for EGD with biopsies included abdominal pain, bloating, diarrhea, weight loss, poor-weight gain, failure to thrive, positive serology for tissue transglutaminase (TTG) or antiendomysial antibody (EMA), reflux symptoms, and failed treatment with acid suppression therapy. Anesthesia was administered by pediatric anesthesiologists. All EGDs were performed using an EG1840K or EG2730K video endoscope (Pentax Medical, Montvale, NJ). During Endoscopy, esophageal (lower, mid, and upper), gastric (antrum and fundus), and duodenal pinch biopsies were routinely obtained using standard biopsy forceps.

### 2.3. Histology

All biopsies were evaluated by an experienced pediatric pathologist at the Women and Children's Hospital of Buffalo, University at Buffalo [7].

### 2.4. Statistical Analysis

Statistical analysis was performed by a professional statistician at the University at Buffalo, Buffalo, NY. Statistical analyses were performed with SAS statistical software (version 9.12, 2004, NC, USA). The means procedure, frequency procedure, and *t*-tests were used to evaluate the prevalence of esophagitis in the practice and to compare it to the prevalence in the general population. One-way ANOVA was used to evaluate the significance of the variables versus the presence of esophagitis in patients with CD. The differences between study groups were considered significant when the *P* value was <0.05 or when 95% CI did not include 1.0 (equivalent to *P* < 0.05).

## 3. Results

There were 3548 EGD performed between January 1, 2003 and December 31, 2007. Mucosal biopsies were obtained in 2218 EGD and are included in the analysis. Forty nine (2.2%) of those had CD (age 1–18 yrs, mean 9.7 yrs ± SD 5.38). Overall, 19 (39%) of those with CD had esophagitis, 95% CI (0.23–0.51) including one patient that had eosinophilic esophagitis. Of the 2169 patients who did not have CD, 997 (45%) had histologic evidence of esophagitis (56% males, mean age 10.77, range 0.25–24 years) There was no significant difference in esophagitis between the group that had CD and the group that did not. Patient demographics and characteristics are summarized in Table 1. There was no difference between the groups in any of these parameters.

## 4. Discussion

Gastroesophageal reflux and esophagitis are commonly diagnosed in the general pediatric gastroenterology practice and can cause significant morbidity including missed school days and interference with daily activities. 

The prevalence of esophagitis in children with CD has never been examined. Collin et al. [8] showed that 0.9% of adult patients with esophagitis and 0.6% of those with esophageal reflux symptoms had CD. They reported that the prevalence of esophagitis was 5.2% in untreated CD, 5.6% in treated CD, and 27% in symptomatic reflux disease. The reflux symptoms in celiac patients were mild; however, the symptoms were alleviated on a gluten-free diet (GFD). Their conclusion was that patients with reflux esophagitis should not be screened for CD. However, Cuomo et al. [9] showed that adult patients with CD have a higher prevalence of reflux esophagitis than those without CD and suggest that CD may represent a risk factor for the development of reflux esophagitis. Our data showed no difference in the prevalence of esophagitis between those who have CD and those who underwent EGD but did not have CD. Our population is different from the populations reported on by Cuomo and Collin in that our data is limited to children and adolescents. Our data suggests that it is prudent to obtain esophageal biopsies at the time of the diagnostic EGD for CD and to consider repeating esophageal biopsies on those who continue to be symptomatic despite GFD and acid suppression.

It is important to note that there is no data on the prevalence of esophagitis in the general population of children and adolescents. This study shows the prevalence of esophagitis in patients who presented with symptoms that required an EGD. It is expected that these patients would have a higher prevalence of esophagitis than those in the general population. Since the prevalence of esophagitis in patients with CD is statistically similar to the prevalence of esophagitis in patients who did not have CD but patients whose symptoms warranted an EGD, it is reasonable to assume that esophagitis occurs with greater frequency in patients with CD than in the general population.

Since patients with CD did not have to repeat EGD, we do not know if the esophagitis resolved on a GFD. Patients with coexistent CD and esophagitis showed clinical improvement when treated with both acid suppression and GFD. Thus, we were unable to determine if the clinical improvement was from acid suppression, GFD, or both. 

Finally, esophageal biopsies can also exclude eosinophilic esophagitis (EE), a diagnosis that has been on the rise over the past 15–20 years. Although, we only identified one patient with CD who concomitantly had EE, there are several case reports and reviews on the increased prevalence of EE in patients with CD compared to the prevalence of EE in the general population [10, 11].

## 5. Conclusion

There was no difference in the prevalence of esophagitis between children diagnosed with CD at the time of their diagnostic EGD and prevalence of esophagitis in children without CD undergoing EGD. A prospective study to determine whether there is a need to treat the esophagitis with acid suppression therapy or whether the esophagitis heals with the gluten-free diet is warranted.

## Figures and Tables

**Table 1:** Patient demographics.

	Celiac disease population *n* = 49				Endoscopy with biopsy population *n* = 2218			
	Number (%)	Mean	Range	SD ±	Number (%)	Mean	Range	SD ±
Number	**49**				**2218**			
Age (y)		9.7	1–18	5.38		10.51	0.25–24	5.68
Gender								
Males	23 (47)				1097 (49)			
Age (Y)		9.39	1–18	5.63		9.55	0.25–20	5.34
Esophagitis	7/49 (14)							
Females	26 (53)				1121 (51)			
Age (Y)		9.98	1–18	5.24		11.46	0.25–24	5.84
Esophagitis	12/49 (24)							

*Esophagitis Present*	**19 (39)**	10.09	1–17	4.98	**997 (45)**	10.77	0.25–24	5.55

Gender								
Male	7/19 (37)				560 (56)			
Age (Y)		9.37	2–14	3.98		10.27	0.25–22	5.54
Female	12/19 (63)				437 (44)			
Age (Y)		12.18	5–18	3.85		11.42	0.25–24	5.49

*Esophagitis absent*	**30 (61)**	9.56	1–18	**5.9**	**1221 (55)**	10.31	0.25–24	5.76

Gender								
Male	16/30 (53)				537 (44)			
Age (Y)		9.4	1–18	6.34		8.84	0.25–24	6.04
Female	14/30 (47)				684 (56)			
Age (Y)		7.95	1–17	5.66		11.49	0.25–20	5.24
